# FGF7 and FGF10 Promote Fate Transition of Human Epidermal Cell-derived Organoids to an Eccrine Gland Phenotype

**DOI:** 10.7150/ijbs.97422

**Published:** 2024-08-01

**Authors:** Junhong Zhao, Lei Zhang, Yonghong Zhang, Manxiu Cao, Cangyu Wang, Anqi Hu, Leilei Cao, Qizhi Luo, Zhen You, Xueping Ma, Liang Gong, Cuiping Zhang, Haihong Li

**Affiliations:** 1 Laboratory of Wound Repair and Dermatologic Surgery, Taihe Hospital, Hubei University of Medicine, Shiyan, Hubei Province, China.; 2 Department of Psychiatry and Clinical Psychology, the Seventh Affiliated Hospital of Sun Yat-sen University, Shenzhen, Guangdong Province, China.; 3 School of Basic Medicine, Academy of Bio-Medicine Research, Hubei Key Laboratory of Embryonic Stem Cell Research, Hubei University of Medicine, Shiyan, Hubei Province, China.; 4 Department of Burns and Plastic Surgery, the Seventh Affiliated Hospital of Sun Yat-sen University, Shenzhen, Guangdong Province, China.; 5 Department of Urology of The First Affiliated Hospital, Zhejiang University School of Medicine, Hangzhou, China.; 6 Liangzhu Laboratory, Zhejiang University Medical Center, Hangzhou, China.; 7 Research Center for Tissue Repair and Regeneration affiliated to the Medical Innovation Research Department and Fourth Medical Center of PLA General Hospital, Beijing, China.

**Keywords:** Epidermal cells, Eccrine sweat glands, Hair follicles, Fibroblast growth factor, Fibroblast growth factor receptor, mitogen-activated protein kinase

## Abstract

**Rationale:** Reconstruction of hair follicles (HFs) and eccrine sweat glands (ESGs) is essential for functional skin regeneration. In skin reconstruction research, we found that foreskin-derived epidermal cells reconstructed HF organoids unidirectionally, but not ESG organoids.

**Methods:** To investigate key genes and pathways influencing the fate of ESG and HF, a transcriptome profiling of ESG placode-containing skin and HF placode-containing skin was employed, and key DEGs were identified and validated by RT-qPCR and immunofluorescence staining in mice and rats. Subsequently, adult human epidermal cell-derived organoids were reconstructed to probe functional roles and mechanisms of FGF7 and FGF10 by series of approaches integrating RT-qPCR, immunofluorescence-staining, WB, apoptosis assay, and pathway interference assay.

**Results:** All members of FGF7 subfamily were among the key DEGs screened, the differential expression of FGF7 and FGF10 and their receptors FGFR1/FGFR2 was verified between ESG placode-containing skin and HF placode-containing skin. *In vivo* and *in vitro* Matrigel plug models showed that both FGF7 and FGF10 promoted fate transition of human epidermal cell-derived organoids to ESG phenotype organoids, FGF7 and FGF10 had a synergistic effect, and mainly function through the FGFR1/2-MEK1/2-ERK1/2 pathway.

**Conclusions:** Adult epidermal cells can be manipulated to reconstruct personalized HF and ESG to meet different needs.

## Introduction

In humans, eccrine sweat glands (ESGs) are an important dynamic thermoregulatory organ to secrete sweat 1. Sweat secretion and evaporation can dissipate the heat generated by physical activities or hot ambient environment, thereby preventing human body from hyperthermia 2, 3. The number of ESGs is fixating in an adult person, which means that new ESGs would not generate after birth, or hardly regenerate after damage 4, 5. Our previous studies have shown ESGCs can reconstitute ESG-organoids in Matrigel, supporting that there are abundant stem cells residing in ESGs 6-10. However, sweating cannot be restored post-severe extensive burns, due to no available ESGCs remain. How to regenerate ESGs remains an urgent challenge.

During embryogenesis, skin appendages including hair follicles (HFs), sweat glands, mammary glands, and salivary glands are all originated from epidermal progenitors 11, 12. These progenitor cells grow downward to form appendage placodes and germs before developing into different skin appendages 4, 12. HFs and ESGs are the two major skin appendages that share many common features 13. Although most animals confine HFs and ESGs to distinct body regions, HFs and ESGs are dual-presenting in nearly all human skin 13, 14.

Three-dimensional (3D) culture provides a physiological mimicking model to study cell differentiation and its mechanisms 15. Extracellular matrix (ECM) is a key regulator of normal homeostasis and differentiation. Cells cultured in the laminin-rich ECM Matrigel can restore key microenvironmental cues and reconstitute the 3D structure of the source tissues 16. We previously have showed that ESGCs can reconstitute ESG organoids in the Matrigel plug model, and that the reconstituted ESG organoids exhibit morphology, structure, and function comparable to native ESG 6-10.

Comparative analysis of high-throughput RNA-sequencing (RNA-seq) data from different tissues or developmental stages is versatile to characterize key differentially expressed genes (DEGs) and signal pathways at transcriptome-wide level 17. A study by Lu *et al.* found that fate decisions of ESGs and HFs are within a narrow developmental window and that the differentiation of ESGs and HFs is spatiotemporally antagonistic in rodents 13. The glabrous foreskin was reported to contain none of the skin appendages in neonates and children 18, 19. Therefore, the harvested foreskin is often used to isolate keratinocytes in regenerative studies 18. It has been reported that foreskin-derived keratinocytes can reconstitute HF-like organoids but not ESG-like organoids in Matrigel plug models. Since epidermal stem cells are common progenitor of ESGs and HFs, it is still ambiguous that why foreskin keratinocytes, including epidermal stem cells, differentiate unidirectionally into HFs rather than ESGs in Matrigel? Furthermore, would it be practical to direct the fate of epidermal cell differentiation endogenously or exogenously?

In this study, in order to investigate key genes and signal pathways involved in ESG and HF fate determination, a transcriptome profiling of mouse skin containing ESG placode and HF placode was performed. Key DEGs were identified and validated by Reverse Transcription-quantitative Polymerase Chain Reaction (RT-qPCR) in both mice and rats. Adult human epidermal cell-derived organoids were successfully reconstituted to probe functional roles of fibroblast growth factor (FGF)7 and FGF10 by a set of approaches integrating RT-qPCR, immunofluorescence staining, western blotting (WB), apoptosis assay, and pathway interference assay, suggesting that FGF7 and FGF10 are key players to promote ESG fate determination mainly through the fibroblast growth factor receptor (FGFR)1/2-MEK1/2-ERK1/2 pathway in foreskin keratinocyte-reconstituted organoids. The reconstitution of organoids with HF and ESG fates highlights the potential application of epidermal cells in reconstituting personalized HFs and ESGs.

## Materials and Methods

### Animals

Two-month-old female and male Sprague-Dawley (SD) rats, 6-8-week-old female and male C57BL/6 mice, and two-month-old male athymic nude (NU/NU) mice were purchased from the Beijing Vital River Laboratory Animal Technology Co. Ltd (Beijing, China). All the animals were maintained on a 12 light /12 dark cycle with *ad libitum* access to food and water. The room temperature (RT) was maintained at 22±1℃ with low humidity. All animal experiments were approved by the Medical Animal Care and Use Committee of Hubei University of Medicine (Shiyan, China) following Laboratory Animal Welfare regulations. The ethical review approval number is Hubei Medical College Animal (Fu) No. 2020-Submission 007.

Female rats/mice were caged overnight with males at ratio 3~4:1. Pregnancy was confirmed by the formation of a copulatory plug and the first morning after getting pregnant was designated as embryonic day (E) 0.5. Pregnant female rats/mice were then housed individually. The ventral footpad skin and dorsal back skin from E14.5, E15.5, E16.5, E17.5, E18.5, E19.5 and E20.5 were collected. These samples were divided into two parts: one part was used for RNA-sequencing (only for mice) and RT-qPCR, while the other part was fixed in 4% paraformaldehyde, dehydrated, cleared, embedded in paraffin, and cut into 5 μm-thick sections for histological examination.

### RNA-seq and comparative bioinformatics analysis

Prior to analysis, adapters and low-quality bases were trimmed from RNA sequencing reads using trim_galore (v0.6.8) under flags: -q 20, --length 20, and -stringency 3. Clean reads were mapped to mm10 assembly by STAR (v2.7.10) aligner with parameters: -- alignSJoverhangMin 8, --alignSJDBoverhangMin 10, --alignSJDBoverhangMin 1, and --chimSegmentMin 15 20. Public annotation of mm10 reference genome was fetched from GENCODE vM25. Genes expression matrix was subsequently measured by htseq program (v0.11.1) 21. Read counts were normalized with fragments per kilobase per million mapped fragments (FPKM) for groups comparison. R package DESeq2 was utilized for differentially expressed genes (DEGs) analysis contingent on the expression counts across various tissues and developmental stages. DEGs were defined based on adjusted p-value cutoff = 0.05 and absolute log_2_FoldChange cutoff = 1 22. Functional enrichment analysis of DEGs on over-represented Kyoto Encyclopedia of Genes and Genomes (KEGG) pathways and gene ontology (GO) was conducted using R package gProfileR 23. Raw p-values were adjusted by Benjamini-Hochberg FDR method. The intersection between DEGs and the total genes involved in significant KEGG pathways was calculated. The RNA-seq data produced in this research have been deposited in the NCBIs Gene Expression Omnibus (GSE237102).

### Histological examination

Paraffin sections were utilized for H&E staining and fluorescence immunohistochemistry (IHC) staining, used, while cell smears were used for fluorescence immunocytochemistry (ICC) staining. Isotype control antibody (ab18450, abcam, Cambridge, UK) was employed as negative controls. Staining results were observed using a fluorescence microscope (Olympus BX51, Tokyo, Japan).

For H&E staining, the paraffin sections were routinely dewaxed in xylene, rehydration in graded alcohol, and subsequently stained with hematoxylin, counterstained in alcoholic-eosin, dehydrate with alcohol, cleared in xylene, and mounted with resin. When ICC staining was performed, **t**he harvested cells were adjusted to 2×10^6^ cells/ml and fixed with 4% paraformaldehyde for 30 min. 5 μL of cell suspension was spread on poly-lysine-coated coverslip, and placed into a 24-well tissue culture plate., then incubated with primary antibodies (Table S2) at 4 ^◦^C overnight, followed by incubation with appropriate Alexa fluor 488-labeled secondary antibodies (Table S3) for 1h at RT in the dark. Finally, the coverslips were counterstained with 5μg/ml 4′, 6′-diamidino-2-phenylindole (DAPI; C1005, Beyotime, Shanghai, China) for 2 min, removed from the wells and mounted with anti-fade mounting medium (P0126, Beyotime, Shanghai, China). For IHC staining, the rehydrated paraffin sections were retrieved with hydroxymethyl (Tris)-ethylenediaminetetraacetic acid (EDTA) buffer pH 9.0 (ZSGB-BIO, Beijing, China), followed by blocking of nonspecific sites with normal goat serum (ZSGB-BIO, Beijing, China). Subsequently, the sections were incubated with primary antibodies (Table S2) and appropriate fluorescein-conjugated secondary antibodies (Table S3). Finally, the sections were counterstained with DAPI and mounted with anti-fade mounting medium.

### Cell isolation and culture

**Figure 1:**
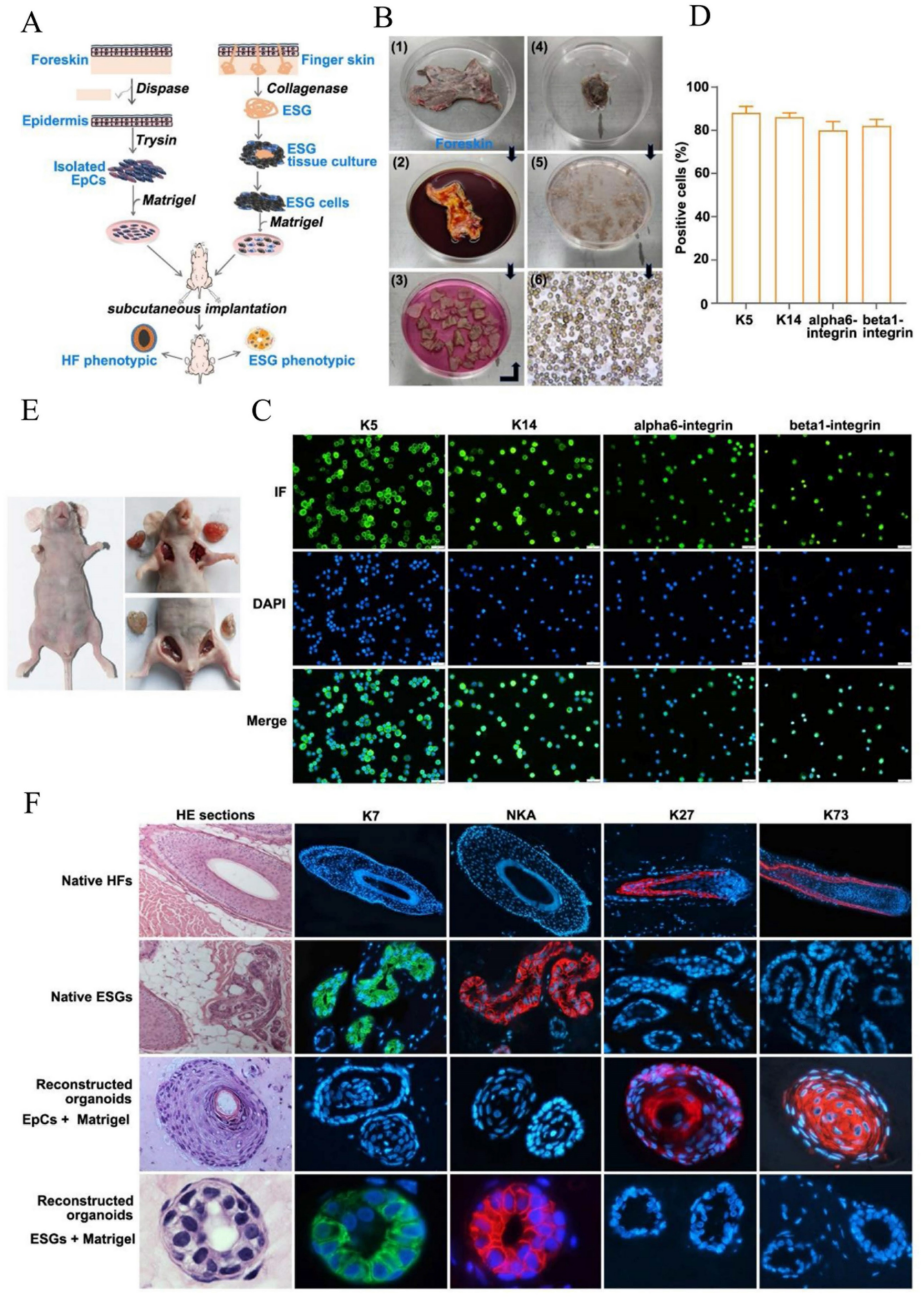
** Epidermal cells reconstruct HF-phenotype organoids in the* in vivo* Matrigel plug model. (A)** Schematic diagram of "FDKCs + Matrigel" and "ESGCs+ Matrigel" to reconstruct 3D organoids. **(B)** Procedure for isolation of FDKCs. **(C)** The isolated EpCs were identified by fluorescent ICC staining using epidermal stem cell markers K5, K14, alpha6-integrin and beta1-integrin. **(D)** The percentage of FDKCs expressing epidermal stem cell markers. The percentage of FDKCs expressing K5, K14, alpha6-integrin and beta1-integrin exceeds 80%. **(E)** Representative implantation images of the *in vivo* Matrigel plug model. "ESGCs + Matrigel" (upper right) and "FDKCs + Matrigel" (lower right) were implanted into the axillary/inguinal fat pads of nude mice, respectively. **(F)** Antigen expression and characterization of constituted organoids and native HFs and ESGs. The organoids constituted by "ESGCs + Matrigel" showed ESG-like structures, and were positive for ESG markers K7 and NKA, but negative for HF markers K27 and K73. The organoids constituted by "FDKCs + Matrigel" displayed HF-like structures, and were positive for HF markers K27 and K73, but negative for ESG markers K7 and NKA.

**Isolation of keratinocytes derived from human foreskin.** (Figure 1A). The isolation procedures for human foreskin-derived keratinocytes (FDKCs) were as previously reported 24, 25. Foreskin specimens were obtained from male children ages of 3 to 10 years old admitted for circumcision in the Urology Department of Taihe Hospital of Hubei University of Medicine (Shiyan, Hubei, China).. Fresh foreskin specimens were rinsed with PBS and removed the subcutaneous fat, then cut into small pieces of 1×1 cm^2^, incubated in 0.2 mg/ml dispase overnight at 4°C, with the dermal side up. After incubation, the epidermis was separated from the dermis, minced, and digested with 0.25% trypsin-0.02% EDTA for 15 min in a carbon dioxide incubator, followed by the addition of Dulbecco's modified Eagle's medium (DMEM) supplemented with 10% fetal bovine serum (FBS; Gibco, MA, USA) to stop digestion. The cell suspension was re-suspended to release the keratinocytes and filtrated through 100 µm and 40 µm mesh cell strainers (Biologix Group Limited, Jinan, China.), then inoculated to collagen IV-coated dishes for 15 min, unattached cells were gently removed and attached FDKCs were harvested 26.

**Human eccrine sweat gland cells (ESGCs) culture** (Figure 1A)**.** Isolation and culture methods of human ESG tissues were as we reported previously 6, 7. Briefly, full-thickness skin specimens used to isolate ESGs were obtained from patients aged between 4 and 16 years old undergoing polydactylyectomy. The finger skin contains only ESGs but no HFs. Subcutaneous fat was removed and the skin specimens were rinsed with Hank's balanced salt solution (Gibco, USA) supplemented with 100 IU/ml penicillin and 100 μg/ml streptomycin. The skin specimens were minced as fine as possible and then were incubated with 2 mg/ml collagenase type II (Gibco, USA) at 37°C for 1-2 hours. ESGs were released from peripheral connective tissues and isolated under an inverted phase contrast microscope (Leica, Germany). The isolated ESGs were then cultured in defined-keratinocyte serum free medium (KSFM, Gibco, USA) with growth supplements at 37°C in a humidified atmosphere of 5% CO_2_/95% air. Medium was refreshed every 2 to 3 days. Upon reaching 70% confluence, the residual ESG tissues were eliminated, and the ESGCs were collected. All human skin specimens obtained informed consent from patients or their guardians and ethical approval from the Ethics Committee of Taihe Hospital (Shiyan, China).

### Matrigel plug models

***In vivo* Matrigel plug assay.** FDKCs or ESGCs (1×10^6^ cells/150 μl DMEM) were mixed with 150 μl concentrated Matrigel (354248, BD Bioscience, USA) on ice. Mice were anesthetized with 1% pentobarbital (8 μl/g weight) prior to the injection of 300 μl of cells/Matrigel mixtures into the axillary/inguinal fat pads of nude mice to examining the effects of FGF7 and FGF10 on epidermal cell-reconstituted organoids *in vivo*. Specifically, 10ng Polyhedrin Delivery System (PODS)-human FGF7 (PODS-FGF7) (PPH187, Cell Guidance System, Cambridge, UK) and 10ng PODS-human FGF10 (PODS-FGF10) (PPH183, Cell Guidance System, Cambridge, UK) were added to the FDKCs/Matrigel mixtures. After 4 weeks, the mice were euthanized and the Matrigel plugs were extracted for histological examination and TUNEL apoptosis assay following embedding in paraffin, or store at -80 °C for WB and RT-qPCR analysis. The primers for RT-qPCR and the antibodies used in histological examination and WB were summarized in Table S1.

***In vitro* Matrigel plug assay.** The FDKCs (5×10^5^) were suspended in 100 μl of KSFM and combined with 100 μl of Matrigel (354234, BD Bioscience, USA) on ice. The mixtures were then plated onto six-well plates and incubated in CO2 humidified incubator for 30 min. Once the mixture had solidified, KSFM complete medium was introduced. In an *in vitro* experiment to investigate the effects of FGF7 and FGF10 on FDKCs-reconstituted organoids, 10 ng/ml of human FGF7 (251-KG; R&D Systems, Inc., Minneapolis, MN, USA) and 10 ng/ml of human FGF10 (345-FG; R&D Systems, Inc., Minneapolis, MN, USA) were separately added to the KSFM complete medium. For the pathway interference assay experiment, 40 μM MEK1/2 specific inhibitor U0126 (S1901, Beyotime, Shanghai, China), 50 μM FGFR1 inhibitor PD166866 (S8493; Selleck,) and 50 μM FGFR2 inhibitor Formononetin (HY-N0183; MCE) were added to KSFM complete medium in the presence or absence of human FGF7 and FGF10, respectively. Cell images were captured at different time points using a phase contrast microscope (Leica, Germany). The culture medium was refreshed every other day. After 14 days, the medium was discarded and the cell cultures were treated with BD cell recovery solution (354253, BD, USA) on ice for 2 h to dissolve the Matrigel, and then centrifuged at 1000 rpm for 5 min at 4 °C. After centrifugation, the supernatant was discarded and the cell pellets were collected for WB and RT-qPCR analysis. Details regarding the primers for RT-qPCR and antibodies used in WB were summarized in Table S1 and S2.

### WB assay

For WB detection, cell pellets were homogenized in cell lysis buffer (P0013, Beyotime, Jiangsu, China). Proteins (50 μg/lane) were separated by 4-20 % sodium dodecyl sulfate-polyacrylamide gel electrophoresis (SDS-PAGE) and transferred onto polyvinylidene fluoride (PVDF) membranes. After incubated in 5% skin milk to block the non-specific sites, the membranes were incubated with the primary antibodies against phosphor-extracellular regulated protein kinases (ERK), P-ERK, JNK (c-Jun N-terminal kinase), P-JNK, p38, P-p38, FGFR1, FGFR2 and glyceraldehyde-3-phosphate dehydrogenase (GADPH), respectively (data on the primary antibodies are described in Table S2) at 4 °C overnight, and then with appropriate horseradish peroxidase (HRP)-labeled secondary antibodies (Table S3) for 1 h at RT. Finally, the protein blot was visualized using ECL plus reagent (P1060, Lablead, Beijing, China) and exposed to X-ray film (Koda, USA). TBS containing 0.1 % Tween-20 (TBS-T) was used for rinsing between steps.

### RT-qPCR

The screened hub genes were confirmed by RT-qPCR. Total RNA was extracted from E19.5-ventral hind foot skin and E17.5-dorsal back skin using RNA isolater Total RNA Extraction Reagent (Vazyme, China). 2μg of RNA per sample were used to generate cDNA using the SuperScript III First-Strand Synthesis Supermix (Vazyme, China). RT-qPCR was performed on an Applied Biosystems QuantStudio Real-Time PCR system (life technologies, USA) using 2× Taq Pro Universal SYBR qPCR Master Mix (Vazyme, China). The amplification procedures were held at 95 °C for 30 sec initially, followed by 40 cycles of 95 °C for 15 s and 60 °C for 1 min. Gene expression analysis was performed with three biological replicates, and quantitative gene expression levels were normalized to the expression of GAPDH using the 2-ΔΔCt method. The primers are listed in Table S1.

### TUNEL apoptosis assay

Apoptotic cells were detected using TUNEL Apoptosis Assay (C1086, Beyotime, Jiangsu, China). TUNEL-positive cells were labeled with green fluorescence, while nuclei were counterstained with blue. Apoptosis quantification was conducted by analyzing300 nuclei from at least 8 random microscopic fields. The total number of apoptotic cells in each section was summed and expressed as a percentage of the total cell number.

### Statistical analysis

Statistical analysis was performed using SPSS version 13.5 for Windows (SPSS Inc., Chicago, IL, USA), with data presented as mean ± standard deviation (SD). Group comparisons were assessed using one-way analysis of variance (ANOVA), followed by least significant difference *t* test with significance determined at *p*-value ≤ 0.05.

## Results

### FDKCs prone to form HF-phenotypic organoids in the *in vivo* Matrigel plug model

H&E staining showed that there was absence of HFs, ESGs and sebaceous glands in foreskin (Figure S1). An *in vivo* mouse Matrigel plug model was used to reconstitute organoids by implanting human FDKCs and human ESGCs, respectively (Figure 1A-E). With the assistance of fluorescent ICC staining, epidermal stem cells were identified using their specific markers, K5, K14, αlpha6-integrin, and beta1-integrin (Figure 1C). The positive rate of all these stem cell markers was all above 80%, indicating that most of the isolated FDKCs are stem cells (Figure 1D). H&E staining showed that the reconstituted organoids of "FDKCs + Matrigel" exhibited a typical HF morphological structure, whereas the reconstituted organoids of "ESGCs + Matrigel" had a typical ESG structure (Figure 1F). As expected, the reconstituted organoids of "ESGCs + Matrigel" were positive with ESG markers K7 and Na^+^-K^+^-ATPase (NKA) but negative with HF markers K27 and K73; otherwise, the reconstituted organoids of "FDKCs + Matrigel" were positive with HF markers K27 and K73, but negative with ESG markers K7 and NKA. Notably, these data provide evidence that FDKCs prone to form HF-phenotypic organoids in the *in vivo* Matrigel plug model (Figure 1A, 1F).

### Transcriptome analysis and histological dissection reveal key determinants in the differentiation of ESGs and HFs

**Figure 2:**
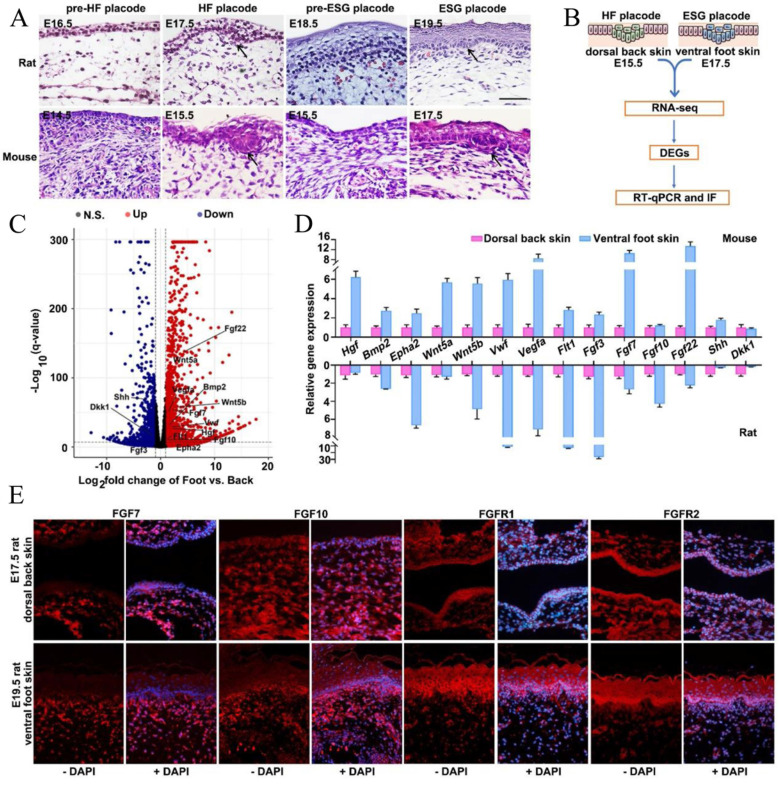
** Transcriptome analysis and histological verification of key determinants during ESG differentiation. (A)** H&E staining showing morphogenesis of ESG placodes (arrow) and HF placodes (arrow) in rats and mice, respectively. In rats, ESG placodes appeared at E19.5 and HF placodes appeared at E17.5. In mice, ESG placodes appeared at E17.5 and HF placodes appeared at E15.5. **(B)** Schematic diagram of DEGs identification by RNA-seq and their validations by RT-qPCR and IF in mouse E17.5 ventral foot skin (containing ESG placodes) and mouse E15.5 dorsal back skin (containing HF placodes). **(C)** Volcano plot of DEGs. The red dots represent significantly upregulated genes, and the blue dots represent significantly downregulated genes. **(D)** RT-qPCR validation of 14 representative genes in both rats and mice samples.** (E)** Fluorescent immunohistochemistry staining of FGF7, FGF10 and FGFR1/2 in rat E19.5 ventral foot skin and E17.5 dorsal back skin. FGF7 and FGF10 were mainly expressed in mesenchyme, and FGFR1/2 were expressed in both epithelium and mesenchyme. The expression intensity of FGF7, FGF10 and FGFR1/2 in rat E19.5 ventral foot skin was higher than that in rat E17.5 dorsal back skin.

Previous studies have demonstrated that ESGs were exclusively observed in rodent ventral foot skin, whereas HFs present only in dorsal back skin, suggesting that ESGs and HFs experience divergent developmental fates 14, 27. As such, we histologically dissected the HF placodes and ESG placodes in both rats and mice. Consistently, we observed that HF placodes were observed on the dorsal skin as early as on E17.5 in rats and E15.5 in mice, respectively; ESG placodes were observed on the ventral foot skin until E19.5 in rats and E17.5 in mice, confirming their divergent fate determination (Figure 2A). Next, tissues from E17.5-mouse ventral foot skin and E15.5-mouse dorsal back skin were collected separately for RNA-seq analysis to identify key genes that are correlated with the early fate determination of these two appendages (Figure 2B). As shown, 12 957 DEGs (log2<0, *p*<0.05) in total were identified. Compared with E15.5 mouse dorsal back skin, there were 6519 up-regulated and 6438 down-regulated genes in E17.5 mouse ventral foot skin (Figure 2C). Agreed with KEGG enrichment assay, GO-enrichment assay suggested 78 upregulated and 25 common downregulated DEGs are involved in development and differentiation related pathways including particularly FGF signaling, namely FGF3, FGF7, FGF10, and FGF22 (Figure 2C, Figure S2). It is worth noting that these four FGFs belong to a same subclass in the FGF family 28. As expected, the expression levels of several known ESG regulators Wnt5a, Wnt5b, sonic hedgehog (SHH), hepatocyte growth factor (HGF), and bone morphogenetic protein 2 (BMP2) were profoundly changed (Figure 2C, Figure S2). Accordingly, RT-qPCR was employed to validate the expression changes of these above components in both mouse and rat tissues (Figure 2D). Given that FGF signaling plays an important role in skin appendage development, that FGF7, FGF10, and FGF22 belong to the FGF7 subfamily, and that both FGF7 and FGF10 are keratinocyte growth factors that function in glandular development, we first focused on FGF7 and FGF10 28. Fluorescent immunohistochemistry (IHC) staining was used to show that all these four proteins were positively seen in the mesenchyme. The dominant expression region of FGF7 and FGF10 were in the mesenchyme, while FGFR1 and FGFR2 were accumulating in both epithelium and mesenchyme with a slight dominance in the epithelium (Fig. 2E). Notably, the expression intensity of FGF7, FGF10, FGFR1 and FGFR2 seem to be greater in ESG placode-containing ventral foot skin than that in HF placode-containing dorsal back skin (Fig. 2E).

### FGF7 and FGF10 promote FDKCs to reconstitute organoids with ESG fate in the* in vivo* Matrigel plug model

**Figure 3:**
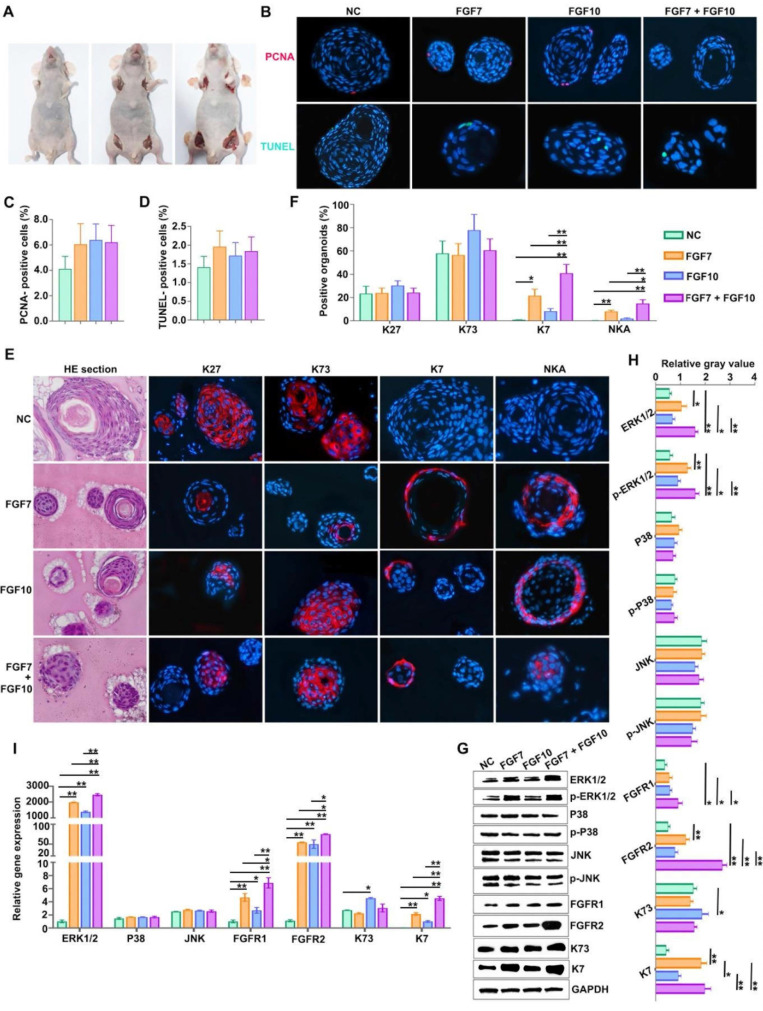
** Addition of FGF7 and FGF10 promotes human epidermal cells to reconstruct ESG-characterized organoids in the *in vivo* Matrigel plug model. (A)** *In vivo* Matrigel plug model. "EpCs + Matrigel" were implanted into the axillary/inguinal fat pads of nude mice in the presence or absence of FGF7-PODS, FGF10-PODS, and FGF7-PODS + FGF10-PODS, respectively. **(B)** Cell proliferation assay and cell apoptosis assay. Upper panel, PCNA fluorescent IHC staining (red staining; showing cell proliferation). Lower panel, TUNEL fluorescent detection (green staining; showing cell apoptosis). **(C)** The percentage of PCNA-positive cells related to figure B. There was no difference among the four groups. **(D)** The percentage of TUNEL-positive cells related to figure b. There was no difference among the four groups. **(E)** Observation of organoids structures and their fate detection using markers. H&E staining shows organoids structures. Fluorescent IHC staining using ESG markers K7 and NKA, and HF markers K27 and K73, respectively. **(F)** Percentage of K7, K27, K73, and NKA positive organoids according to the IHC staining. The percentage of organoids with ESG phenotypes (K7, NKA) was higher in the presence of FGF7 and FGF10 alone and in combination compared to the NC group. **(G)** WB detection of the protein expression of ERK1/2, p-ERK1/2, P38, p-P38, JNK, p-JNK, FGFR1/2, ESG marker K7, HF marker K73, and internal control GAPDH. **(H)** The relative integrated density of ERK1/2, p-ERK1/2, P38, p-P38, JNK, p-JNK, FGFR1/2, K7, and K73. **(I)** RT-qPCR analysis mRNA expression levels of ERK1/2, P38, JNK, FGFR1/2, K7, and K73. The asterisk (*) indicate significant differences at* p* < 0.05, and the asterisks (**) indicate significant differences at *p* < 0.001 (one-way ANOVA,* t* test).

As mentioned above, FGF7 and FGF10 may act as key players in differentiation of skin appendages. To probe potential determinants in FDKCs-reconstituted organoids, we applied exogenous feeding experiments in the *in vivo* Matrigel plug model to monitor the effects of FGF7 and FGF10 (Figure 3A). In the normal control (NC) group, the percentages of organoids positive for HF markers K27 and K73 were 23.44±6.31% and 57.89±10.63%, respectively; in contrast, the percentages of organoids positive for ESG markers K7 and NKA were extremely low (Figure 3E, 3F). In the FGF7 feeding group, the percentage of organoids positive for ESG markers K7 and NKA rise up to 21.62±5.61% and 8.13±1.15%, respectively (Figure 3E, 3F). In the FGF10 feeding group, the percentage of organoids positive for ESG markers K7 and NKA rise up to 8.33±2.31% and 2.03±0.61%, respectively; meanwhile, the positive percentage of organoids for HF markers K27 (30.38±4.06%) and K73 (77.85±13.56%) increased slightly, but there was no significant difference compared with the NC group (Figure 3E, 3F).

In the FGF7/10-combined feeding group, a drastic increase in the percentage of organoids positive for ESG markers K7 (40.85±7.60%) and NKA (14.93±3.32%) were obtained, indicating that when used in combination, FGF7 and FGF10 additively promote FDKC to reconstruct organoids with ESG characteristics (Figure 3E, 3F). Interestingly, IHC results showed that FGF7 and FGF10 had no effects on the proliferation [(F(3, 8)=1.9, *p*>0.05] or apoptosis [(F(3, 8)=1.270, *p*>0.05] of the organoids reconstituted in Matrigel (Figure 3B, 3C, 3D), but exhibited a significant pro-differentiation effect (Figure 3F). The mRNA and protein expression levels of ESG marker K7 in the FGF7, FGF10 and FGF7/10 groups were generally higher than those in the NC group, with the FGF7/10 group having the highest expression level (Figure 3G-I). On the other hand, FGF7 had no effect on the expression level of K73, and FGF10 had a slight but significant promoting effect (Figure 3G-I).

### FGF7 and FGF10 promote FDKCs to reconstitute organoids with ESG fate in the *in vitro* Matrigel plug model

**Figure 4:**
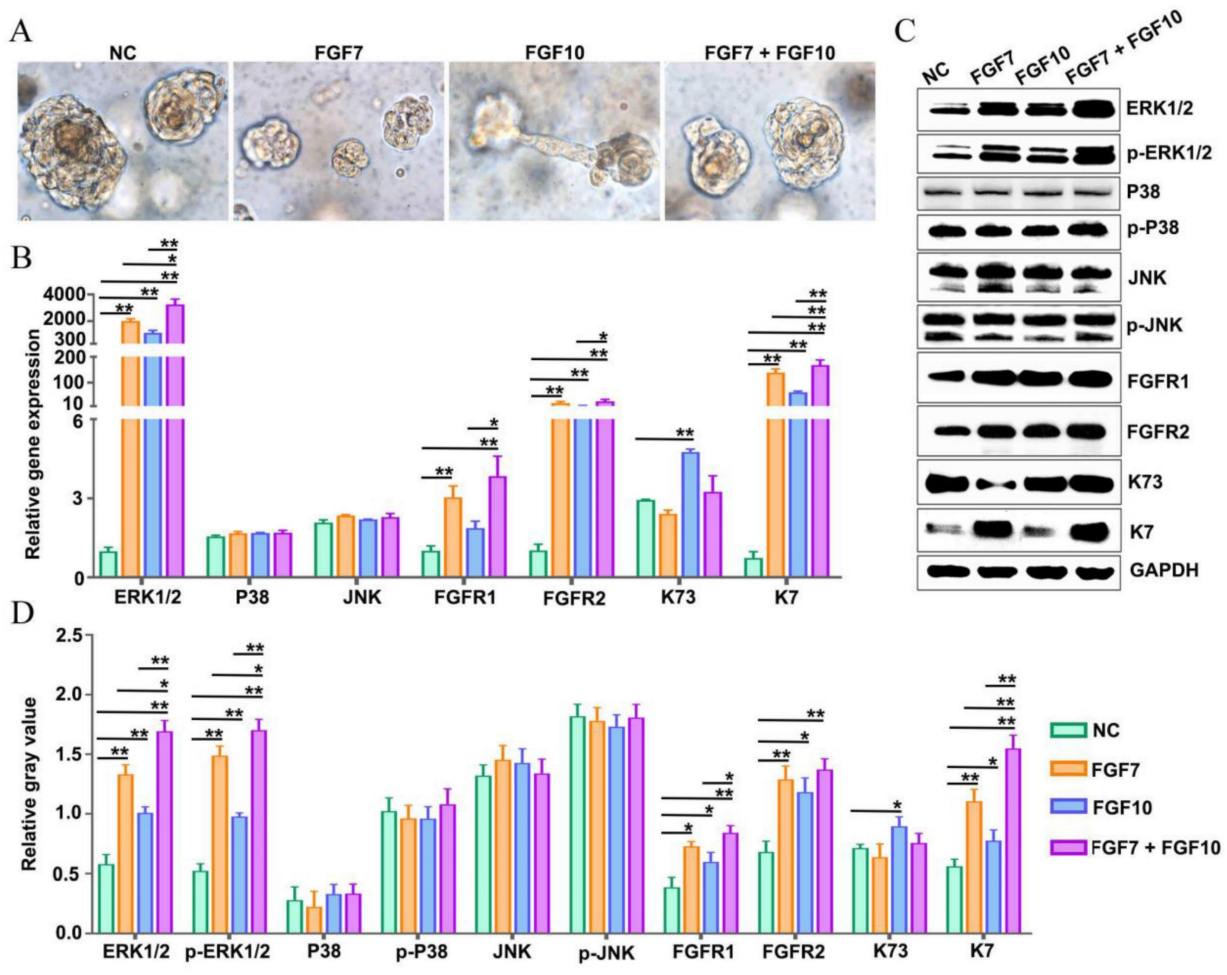
** Addition of FGF7 and FGF10 stimulates human FDKCs to reconstitute ESG-characterized organoids in the *in vitro* Matrigel plug model. (A)** Phase contrast images showing the morphology of organoids constructed with "FDKCs + Matrigel" in the presence and absence of FGF7 and FGF10 at day 9 in the *in vitro* Matrigel plug model. **(B)** RT-qPCR detection of transcriptional expression of ERK1/2, P38, JNK, FGFR1/2, K7, and K73. **(C)** WB detection of the protein expression of ERK1/2, p-ERK1/2, P38, p-P38, JNK, p-JNK, FGFR1/2, ESG marker K7, and HF marker K73 and internal control GAPDH. **(D)** The relative integrated density of ERK1/2, p-ERK1/2, P38, p-P38, JNK, p-JNK, FGFR1/2, K7, and K73. The asterisk (*) indicate significant differences at* p* < 0.05, and the asterisks (**) indicate significant differences at *p* < 0.001 (one-way ANOVA,* t* test).

The* in vitro* Matrigel plug model showed that FDKCs formed small cell clusters 2-3 days after seeding the mixture of FDKCs and Matrigel (Figure S3). The size of the cell clusters increased over time (Figure S3). Nine days after seeding, some organoids were connected to other organoids through tubular structures in the FGF10-fed group (Figure 4A). However, the phenomenon was not shown in the NC group, FGF7 group, or FGF7/10 group (Figure 4A).

The mRNA and protein expression profiling of K7 and K73 in the *in vitro* Matrigel plug model was consistent with that in the *in vivo* Matrigel plug model (Figure 3, 4). There were significant differences in the mRNA and protein expression levels of K7. In comparison with the NC group, the mRNA and protein expression levels of K7 in the FGF7, FGF10 and FGF7/10 groups were significantly higher, with the greatest level in the FGF7/10 combined feeding group (Figure 4B-D). It is explicated that FGF10 had no effect on the expression level of K73 (Figure 3, 4).

### FGFR1 and FGFR2 inhibitors reduce reconstituted organoids with ESG fate

**Figure 5:**
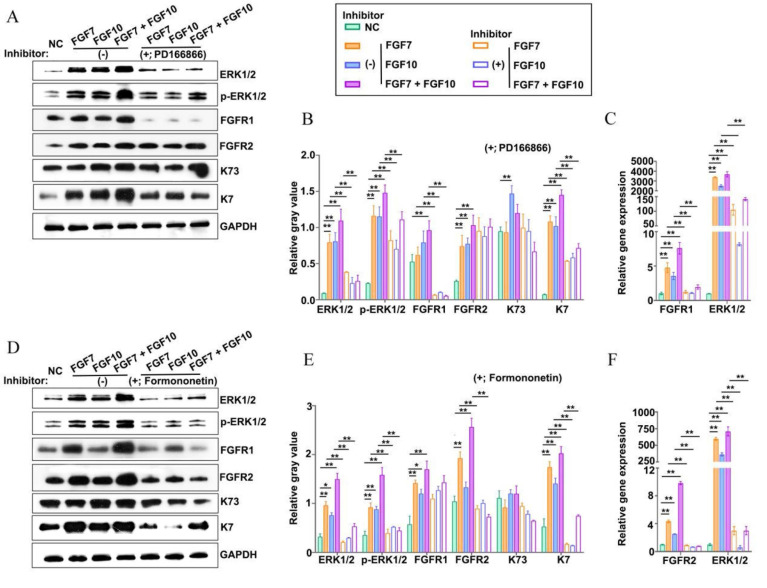
** Addition of inhibitors targeting FGFR1 or FGFR2 impairs reconstitution of FDKCs into ESG-characterized organoids. (A-C)** Protein and mRNA expression levels of ERK1/2, p-ERK1/2, FGFR1, FGFR2, K7, and K73 in FGF- fed organoids with (+) or without (-) the addition of the FGFR1 inhibitor PD166866. **(A)** WB assay for protein expression levels. GAPDH serves as the internal control. **(B)** The relative integrated density of ERK1/2, p-ERK1/2, FGFR1, FGFR2, K7, and K73 in accordance with (A). **(C)** RT-qPCR detection of transcriptional expression levels. **(D-F)** Protein and mRNA expression levels of ERK1/2, p-ERK1/2, FGFR1, FGFR2, K7, and K73 in FGF- fed organoids with (+) or without (-) the addition of the FGFR2 inhibitor formononetin. **(D)** WB assay for protein expression levels. GAPDH serves as the internal control. **(E)** The relative integrated density of ERK1/2, p-ERK1/2, FGFR1, FGFR2, K7, and K73 in accordance with (D). **(F)** RT-qPCR detection of transcriptional expression levels. The asterisk (*) indicate significant differences at* p* < 0.05, and the asterisks (**) indicate significant differences at *p* < 0.001 (one-way ANOVA,* t* test).

The FGF7 subfamily functions by binding to and activating the high-affinity transmembrane tyrosine kinase receptors FGFR1 and FGFR2 29. To answer whether FGFR1 and FGFR2 were involved in the formation of ESG-characterized organoids stimulated by FGF7 and FGF10 in the *in vitro* Matrigel plug model, FGFR1 and FGFR2 inhibitors were introduced in the culture (Figure 5A-F). The mRNA and protein expression levels of FGFR1 and FGFR2 were determined upon the use of PD166866 (a FGFR1 inhibitor) and formononetin (a FGFR2 inhibitor), respectively, to confirm the specificity of the drugs (Figure 5A-F) 30-32. Both the two inhibitors reduced the mRNA expression level of ESG marker K7, with a more profound effect of formononetin than that of PD166866, implying that FGFR2 played a dominant role in the reconstitution of ESG-characterized organoids from FDKCs (Figure 5A-F).

### MEK1/2-specific inhibitor U0126 compromised FDKCs to reconstitute ESG-characterized organoids

**Figure 6:**
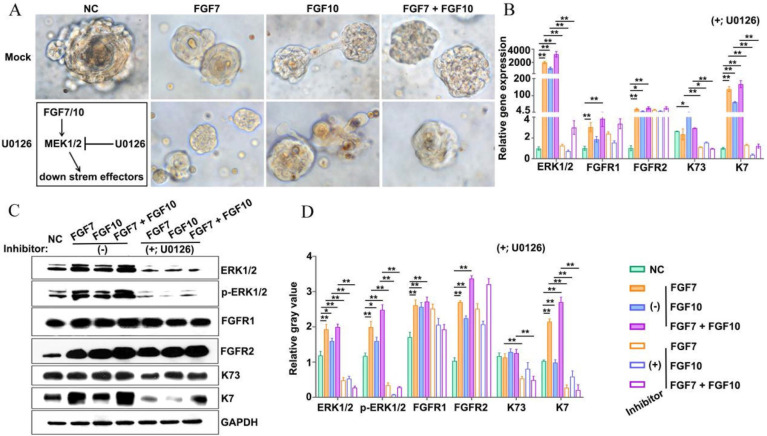
** Addition of inhibitors targeting MEK1/2 impairs reconstitution of FDKCs into ESG-characterized organoids. (A)** Phase-contrast images show the morphology of 3D organoids constructed by "FDKCs + Matrigel" in the presence of FGF7 and FGF10 with or without the MEK1/2 inhibitor U0126 addition at day 9 of culture.** (B)** RT-qPCR detection of transcriptional expression levels of ERK1/2, FGFR1, FGFR2, K7, and K73 in FGF-fed organoids with (+) or without (-) the addition of the MEK1/2 inhibitor U0126. **(C, D)** Protein expression of ERK1/2, p-ERK1/2, FGFR1, FGFR2, K7, and K73 in FGF- fed organoids with (+) or without (-) the addition of the MEK1/2 inhibitor U0126. GAPDH serves as the internal control. **(C)** WB assay. **(D)** The relative integrated density of ERK1/2, p-ERK1/2, FGFR1, FGFR2, K7, and K73 in accordance with (C). The asterisk (*) indicate significant differences at* p* < 0.05, and the asterisks (**) indicate significant differences at *p* < 0.001 (one-way ANOVA,* t* test).

To test whether the MEK1/2-ERK1/2 pathway was involved in the reconstitution of ESG-characteristic organoids in the presence of FGF7 and FGF10, the MEK1/2 inhibitor U0126 was added to the culture medium of the *in vitro* Matrigel plug assay (Figure 6A). Observed under inverted phase contrast microscopes, there was no obvious difference in the morphology of organoids regardless of the addition of U0126 (Figure 6A). RT-qPCR showed that U0126 decreased the mRNA level of the ESG marker K7 and the HF marker K73 (Figure 6B). Moreover, WB showed that the expression levels of K7 and K73 protein were significantly reduced in the presence of U0126 (Figure 6C, 6D). The evidence suggested that the retardation of MEK1/2-ERK1/2 pathway was supposed to compromise the ability of FDKCs to reconstitute ESG-characterized organoids.

### FGF7 and FGF10 promote FDKCs to reconstitute organoids with ESG fate mainly via the FGFR1/2-MEK1/2-ERK1/2 pathway

In the KEGG analysis, the MAPK-Ras and PI3K-AKT signaling pathways were among the top five enriched signaling pathways, and the MAPK-Ras and PI3K-AKT signaling pathways are also downstream signaling pathways regulated by FGF-FGFR (Figure S2). To probe the underlying mechanisms of FGF7 and FGF10 in promoting FDKCs to reconstitute organoids with ESG fate, the interaction of FGFR1 and FGFR2, and their components of RAS-MAPK and PI3K-AKT were investigated (Figure 2-6, Figure S4).

In the *in vivo* Matrigel plug samples, significant differences were observed in the relative integrated density of ERK1/2 [(F(3, 8)=6.28, *p*<0.05], p-ERK1/2 [(F(3, 8)=3.22, *p*<0.05], FGFR1 [(F(3, 8)=76.04, *p*<0.05], FGFR2 [(F(3, 8)=71.02, *p*<0.05], AKT [(F(3, 8)=8.33, ^p^<0.05], and p-AKT [(F(3, 8)=7.93, *p*<0.05], other than in p38 [(F(3, 8)= 0.389, *p*>0.05], p-p38 [(F(3, 8)= 2.04, *p*>0.05], JNK [(F(3, 8)=0.66, *p*>0.05], and p-JNK [(F(3, 8)=4.01, *p*>0.05], demonstrating that in comparison with that in the NC group, relative integrated densities of ERK, p-ERK, FGFR1 and FGFR2 in FGF feeding groups (FGF7 group, FGF10 group and FGF7/10 group) were significantly higher, with a profound additive effect of FGF7 and FGF10 (*p*<0.05) (Figure 3G, 3H, S1). The relative mRNA expression levels of ERK1/2, FGFR1, and FGFR2 were significantly different among the four groups, with their corresponding NC groups being the lowest (Figure 3I). There were no significant differences in the relative gene expression levels of p38 and JNK among the 4 groups (Figure 3I).

In the *in vitro* Matrigel plug samples, the dynamic changes of relative integrated densities of ERK1/2, p-ERK1/2, p38, p-p38, JNK, p-JNK, AKT, p-AKT, FGFR1, and FGFR2 were consistent with those in the *in vivo* Matrigel plug samples among the 4 groups (Figure 3G, 3H, 4C, 4D). The relative mRNA expression levels of ERK1/2, p38, JNK, FGFR1 and FGFR2 also showed similar trends in the *in vivo* and* in vitro* Matrigel plug samples among the 4 groups (Figure 3I, 4B). The* in vitro* and* in vivo* Matrigel plug assays both recommended that FGF7 and FGF10 promoted FDKCs to reconstitute organoids with ESG fate, accompanying with increase of expression levels of FGFR1, FGFR2 and ERK1/2, supporting that FGFR1, FGFR2 and ERK1/2 were involved in FDKC-reconstituted organoids with ESG fates (Figure 3, 4).

In addition, signal pathway interference assays showed that both inhibitors of FGFR1 and FGFR2 reduced the expression levels of ERK1/2. It was worth noting that formononetin had stronger inhibitory effect than PD166866, implying that FGFR2 might play a dominant role (Figure 5). The MEK1/2 inhibitor U0126 significantly reduced mRNA and protein expression levels of ERK1/2, but had no effect on FGFR1 and FGFR2, indicating that MEK1/2-ERK1/2 was involved in the ESG fate determination and was located downstream of FGFR1/2 (Figure 6).

## Discussion

ESGs are essential for thermoregulation in human 4. In the clinical treatment of severe extensive burns, although the skin is restored well, the skin appendages including ESGs would be failed to regenerate; therefore, the survivors often develop heat intolerance 33. How to obtain source cells to regenerate and repair the destroyed or lost ESGs need to be solved. In addition to ESG-derived adult stem cells, there are three major stem cell types that might be used as source cells for the repair and regeneration of ESGs: embryonic stem cells, induced pluripotent stem cells (iPSCs), and adult stem cells 3, 34. Nowadays, clinical applications of embryonic stem cells and iPSCs are broadly banned due to ethical concerns and potential tumorigenicity 35, 36. Alternatively, adult stem cells serve as the optimal cell sources, mainly include adult mesenchymal stem cells (MSCs) and epidermal stem cells. Mesodermal-derived MSCs had multi-lineage differentiation potential and had been shown to transdifferentiate into ectodermal lung epithelial cells, hepatocytes, HF cells and ESGCs 37. However, the transdifferentiation rate was not as high as expected, and the process was very complicated 37, 38. Epidermal stem cells are the progenitor cells of skin appendages. Therefore, inducing epidermal stem cells to differentiate into ESGs is simpler than inducing MSCs to differentiate into ESGs.

To obtain cues that determine the differentiation of epidermal stem cells into ESGs or HFs, a transcriptome profiling of mouse E17.5-foot ventral skin (containing ESG placodes) and mouse E15.5-dorsal back skin (containing HF placodes) were performed. The FGF7 subfamily was among the screened DEGs. Previous studies based on genome-wide transcriptome analysis showed that mouse ESGs were specified by BMPs and FGFs and were repressed by SHH 13. In the study, we preferred to target FGF signaling. The reasons were as follows. Four FGF family members, FGF3, FGF7, FGF10 and FGF22, were up-regulated in rat/mouse ESG placode-containing skin than in HF placode-containing skin. FGFs were shown to be key molecules of the integrated networks in organogenesis and regenerative medicine 39. The study by Lu *et al.* showed that FGF2, FGF7, and FGF18 were more transcribed in mouse ESG placode-containing skin than in mouse HF placode-containing skin 13. FGF7 was a common DEG screened in our study and the study by Lu and colleagues 13. FGF10 shared 51% homology with FGF7 and were said to have similar biological function, and even since they were considered to be glandular mitogens, and were involved in the growth or branching of mammary glands, submandibular glands and lung 40-43. It was therefore interesting to address whether FGF7 and FGF10 contribute to ESG fate determination.

FGF7 and FGF10 are both secreted by the mesenchyme and functions as a paracrine regulator acting upon epithelium by specifically activating two receptors FGFR1b/FGFR2b 29. There are four downstream signaling pathways regulated by FGF-FGFR, including RAS/mitogen-activated protein kinase (MAPK), phosphatidylinositol-4,5-bisphosphate 3-kinase (PI3K)-protein kinase B (AKT), phospholipase C gamma (PLCγ), and signal transducer and activator of transcription (STAT) 44. In our study, KEGG enrichment analysis suggested that RAS-MAPK and PI3K-AKT pathways were significantly enriched between E17.5-foot ventral skin and E15.5-dorsal back skin, providing practical cues of investing the fate-determining mechanisms of skin appendage organoid regeneration (Figure S2).

First, transcript levels of FGF7 and FGF10 in rat/mouse ESG-placode skin and HF-placode skin suggested a role for FGF7 and FGF10 in the development of skin and skin appendages. FGF7 and FGF10 transcript levels were higher in ESG-placode than in HF-placode, suggesting that ESG placode stage requires higher levels of FGF7 and FGF10 than the HF placode stage. The study by Lu *et al.* also showed that both human dorsal skin transitions temporally from HF to the coexistence of HFs and ESGs, and the development of mouse ESG placodes, the transcript levels of FGFs were elevated 13. Changes in FGF levels suggested that higher levels of FGFs were required for ESG placode development.

Second, the functional roles of FGF7 and FGF10 in FDKC-reconstituted organoids were dissected by exogenous feeding experiments using Matrigel plug models. The isolated FDKCs were heterogeneous cells that were freshly isolated, in theory including epidermal stem cells, transient amplifying-cells and post-mitotic cells 45. The percentage of epidermal stem cell marker-positive cells among FDKCs exceeds 80%, ensuring the proportion and number of stem cells with differentiation capability. Due to short half-life and poor thermal stability *in vitro*, the application of FGFs is always limited 46. For sustained release of FGF in the *in vivo* Matrigel plug model, PODS-FGF7 and PODS-FGF10 were used, as PODS crystals were said to be very stable and provide sustained release of active peptides 47. A previous study had shown that PODS-LIF protein presents at more than 50% of peak levels compared with LIF that was completely vanished at day 6 47. From the perspective, PODS crystals maintained the constant activity of FGFs during our treatments.

Previous studies had shown that FGF7 and FGF10 regulated cell proliferation, survival, and differentiation 48-50. In the study, cell proliferation was detected by ICC assay of PCNA intensity, cell survival was indicated by TUNEL apoptosis assay, and cell fate was examined by fluorescence IHC staining of two ESG markers K7 and NKA, and two HF markers K27 and K73, respectively 51. The results showed that in thick Matrigel, exogenous application of FGF7 and FGF10 had no effect on cell proliferation and apoptosis, but had a significant effect on cell differentiation. In the absence of FGF7 and FGF10, reconstituted organoids tend to had an HF phenotype rather than an ESG phenotype. However, in the presence of FGF7 or FGF10, an increased rate of ESG-characteristic organoids was observed, concomitant with enhanced expressions of K7 and NKA, markers of ESGs, suggesting that FGF7 and FGF10 promoted FDKCs to reconstitute organoids with an ESG phenotype. Previous studies by Xu *et al.* showed that FGF7 induced transdifferentiation of human MSCs into ESGCs, and by Guo *et al.* showed that the overall structure and length of HFs did not appear to be markedly affected in FGF7 knockout mice, indicating that FGF7 play a role in ESGs but not in HFs 52, 53. Reports on the role of FGF10 in hair were inconsistent. FGF10 significantly stimulated HF proliferation in organ cultures and promoted HF growth in C57BL/6 mice, whereas FGF10 was dispensable for HF development in FGF10 knockout mice 54-57. There had been no reports on the role of FGF10 in ESGs. Notably, in our study, the percentage of organoids positive for ESG-markers was highest in all four groups when FGF7 and FGF10 were used in combination. The positive percentage of organoids with ESG phenotype in the FGF7/10 group was significantly higher than that in the FGF7 and FGF10 groups, indicating that FGF7 and FGF10 had synergistic pro-differentiation effects. Previous studies suggested that FGF7 subfamily members elicit non-redundant functions, due to the structural basis harboring variant heparin sulfate binding affinities 58.

Third, the downstream signaling pathways activated by FGF7 and FGF10 were studied. In our study, FGF7 and FGF10 enhanced the expression levels of FGFR1, FGFR2, ERK1/2 and AKT in keratinocyte-reconstituted organoids, although they had little effect on P38 and JNK. Compared with the NC group, the expression level changes of the MAPK-ERK signaling pathway were higher than those of the AKT signaling pathway in the FGF7, FGF10 and FGF7/10 groups, implying that FGF7 and FGF10 promoting FDKC-reconstituted organoids with ESG fate mainly via activating the MAPK-ERK pathway. Signal pathway interference assays further showed that inhibitors of FGFR1, FGFR2 and MEK1/2 partially diminished the effects of FGF7 and FGF10, indicating there were other signals. The relative integrated density of FGFR2 was higher than that of FGFR1, and the FGFR2 inhibitor blocked the expression of ESG-related gene K7 much strong than the FGFR1 inhibitor, collectively suggesting that FGFR2 plays a dominant role. Therefore, FGF7 and FGF10 promote FDKC-reconstituted organoids with ESG fate mainly through the FGFR1/2-MEK1/2-ERK1/2 pathway. It had been reported that the FGFR1/2-MEK1/2-ERK1/2 pathway was pivotal for salivary, lacrimal and submandibular gland regeneration 43, 59, 60. Overall, our study of FDKC-reconstituted organoids with HF-phenotype and/or ESG-phenotype highlights the potential application of epidermal cells in reconstituting personalized HFs and ESGs.

## Supplementary Material

Supplementary figures and tables.

## Figures and Tables

**Figure 7:**
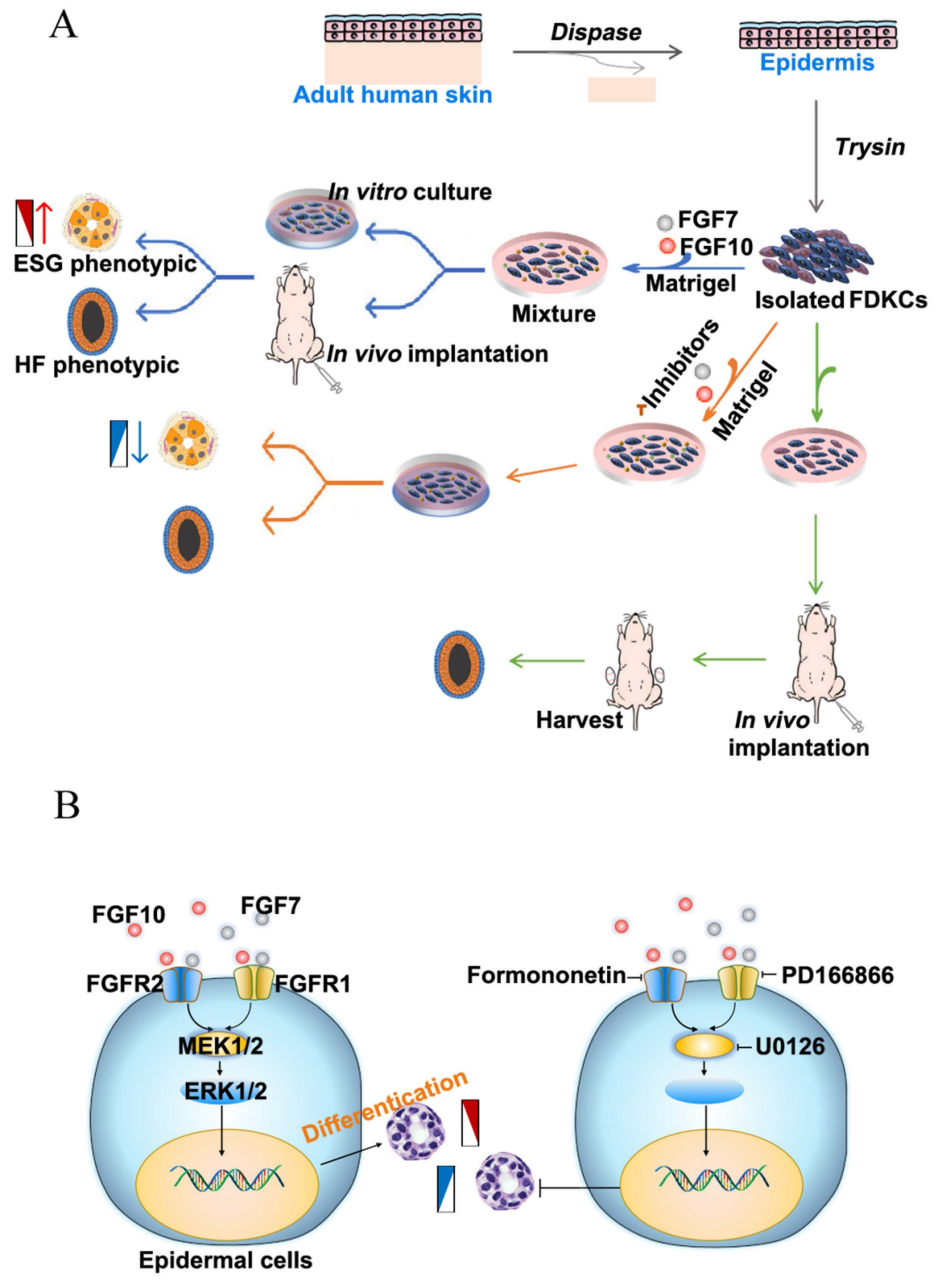
** Flowchart and schematic of the underlying mechan. (A)** Flowchart for the study of the effects of FGF7 and FGF10 on the differentiation of FDKCs. **(B)** Schematic of the underlying mechanisms by which FGF7 and FGF10 promote differentiation of FDKCs into ESG-characterized organoids. Exogenous FGF7/10 binds to FGFR1/2 (mainly FGFR2), which subsequently activates MEK1/2-ERK1/2 signaling and promotes differentiation of FDKCs into ESG-phenotypic organoids. Otherwise, inhibition of the FGFR1/2-MEK1/2-ERK1/2 pathway reduces FDKCs differentiation into ESG-characterized organoids.

## Abbreviations

NKA: Na^+^-K^+^-ATPase
FDKCs: foreskin-derived keratinocytes
ESGs: eccrine sweat glands
ESGCs: eccrine sweat gland cells
HFs: hair follicles
ICC: immunocytochemistry. FDKCs: foreskin-derived keratinocytes
PCNA: proliferating cell nuclear antigen
TUNEL: terminal deoxynucleotidyl transferase (TdT)-mediated dUTP nick end labeling
PODS: polyhedrin delivery system
FGF: fibroblast growth factor
FGFR: fibroblast growth factor receptor

## References

[1] Cui CY, Schlessinger D (2015). Eccrine sweat gland development and sweat secretion. Exp Dermatol.

[2] Cheshire WP, Freeman R (2003). Disorders of sweating. Semin Neurol.

[3] Lin Y, Chen L, Zhang M (2021). Eccrine Sweat Gland and Its Regeneration: Current Status and Future Directions. Front Cell Dev Biol.

[4] Saga K (2002). Structure and function of human sweat glands studied with histochemistry and cytochemistry. Prog Histochem Cytochem.

[5] Montagna W (2012). The structure and function of skin. Elsevier.

[6] Li H, Chen L, Zeng S (2015). Matrigel basement membrane matrix induces eccrine sweat gland cells to reconstitute sweat gland-like structures in nude mice. Exp Cell Res.

[7] Li H, Zhang M, Chen L (2016). Human eccrine sweat gland cells reconstitute polarized spheroids when subcutaneously implanted with Matrigel in nude mice. J Mol Histol.

[8] Li X, Li H, Zhang M (2017). Cell proliferation and differentiation during the three dimensional reconstitution of eccrine sweat glands. J Mol Histol.

[9] Du L, Zhang L, Zhao J (2022). Autophagy, not apoptosis, plays a role in lumen formation of eccrine gland organoids. Chin Med J (Engl).

[10] Zhang M, Li H, Chen L (2018). Three-dimensional reconstructed eccrine sweat glands with vascularization and cholinergic and adrenergic innervation. J Mol Histol.

[11] Fuchs E (2016). Epithelial Skin Biology: Three Decades of Developmental Biology, a Hundred Questions Answered and a Thousand New Ones to Address. Curr Top Dev Biol.

[12] Biggs LC, Mikkola ML (2014). Early inductive events in ectodermal appendage morphogenesis. Semin Cell Dev Biol.

[13] Lu CP, Polak L, Keyes BE (2016). Spatiotemporal antagonism in mesenchymal-epithelial signaling in sweat versus hair fate decision. Science.

[14] Chen Z, Zhao J, Yan Y (2022). Differential distribution and genetic determination of eccrine sweat glands and hair follicles in the volar skin of C57BL/6 mice and SD rats. BMC Vet Res.

[15] Weaver VM, Fischer AH, Peterson OW (1996). The importance of the microenvironment in breast cancer progression: recapitulation of mammary tumorigenesis using a unique human mammary epithelial cell model and a three-dimensional culture assay. Biochem Cell Biol.

[16] Kageyama T, Shimizu A, Anakama R (2022). Reprogramming of three-dimensional microenvironments for in vitro hair follicle induction. Sci Adv.

[17] Hrdlickova R, Toloue M, Tian B (2017). RNA-Seq methods for transcriptome analysis. Wiley Interdiscip Rev RNA.

[18] Higgins CA, Chen JC, Cerise JE (2013). Microenvironmental reprogramming by three-dimensional culture enables dermal papilla cells to induce de novo human hair-follicle growth. Proc Natl Acad Sci U S A.

[19] Taylor JR, Lockwood AP, Taylor AJ (1996). The prepuce: specialized mucosa of the penis and its loss to circumcision. Br J Urol.

[20] Dobin A, Davis CA, Schlesinger F (2013). STAR: ultrafast universal RNA-seq aligner. Bioinformatics.

[21] Anders S, Pyl PT, Huber W (2015). HTSeq-a Python framework to work with high-throughput sequencing data. Bioinformatics.

[22] Love MI, Huber W, Anders S (2014). Moderated estimation of fold change and dispersion for RNA-seq data with DESeq2. Genome Biol.

[23] Kolberg L, Raudvere U, Kuzmin I (2020). gprofiler2 - an R package for gene list functional enrichment analysis and namespace conversion toolset g:Profiler. F1000Res.

[24] McHeik JN, Barrault C, Pedretti N (2015). Study of proliferation and 3D epidermal reconstruction from foreskin, auricular and trunk keratinocytes in children. Burns.

[25] Wang X, Shen C, Li Z (2018). Efficient isolation and high yield of epidermal cells from foreskin biopsies by dynamic trypsinization. Burns.

[26] Stanley JR, Foidart JM, Murray JC (1980). The epidermal cell which selectively adheres to a collagen substrate is the basal cell. J Invest Dermatol.

[27] Kamberov YG, Karlsson EK, Kamberova GL (2015). A genetic basis of variation in eccrine sweat gland and hair follicle density. Proc Natl Acad Sci U S A.

[28] Oulion S, Bertrand S, Escriva H (2012). Evolution of the FGF Gene Family. Int J Evol Biol.

[29] Zinkle A, Mohammadi M (2019). Structural Biology of the FGF7 Subfamily. Front Genet.

[30] Yi L, Lan G, Ju Y (2021). Blockade of Fgfr1 with PD166866 Protects Cartilage from the Catabolic Effects Induced by Interleukin-1β: A Genome-Wide Expression Profiles Analysis. Cartilage.

[31] Wu X, Xu H, Wu Z (2015). Formononetin, a novel FGFR2 inhibitor, potently inhibits angiogenesis and tumor growth in preclinical models. Oncotarget.

[32] Tay KC, Tan LT, Chan CK (2019). Formononetin: A Review of Its Anticancer Potentials and Mechanisms. Front Pharmacol.

[33] Fu X, Sun T, Li X (2005). Morphological and distribution characteristics of sweat glands in hypertrophic scar and their possible effects on sweat gland regeneration. Chin Med J (Engl).

[34] Li H, Fu X (2012). Mechanisms of action of mesenchymal stem cells in cutaneous wound repair and regeneration. Cell Tissue Res.

[35] Lee H, Park J, Forget BG (2009). Induced pluripotent stem cells in regenerative medicine: an argument for continued research on human embryonic stem cells. Regen Med.

[36] De Miguel-Beriain I (2015). The ethics of stem cells revisited. Adv Drug Deliv Rev.

[37] Liu W, Song F, Ren L (2015). The multiple functional roles of mesenchymal stem cells in participating in treating liver diseases. J Cell Mol Med.

[38] Azizi Z, Abbaszadeh R, Sahebnasagh R (2022). Bone marrow mesenchymal stromal cells for diabetes therapy: touch, fuse, and fix?. Stem Cell Res Ther.

[39] Katoh Y, Katoh M (2005). Comparative genomics on FGF7, FGF10, FGF22 orthologs, and identification of fgf25. Int J Mol Med.

[40] Bellusci S, Grindley J, Emoto H (1997). Fibroblast growth factor 10 (FGF10) and branching morphogenesis in the embryonic mouse lung. Development.

[41] Sathi GA, Farahat M, Hara ES (2017). MCSF orchestrates branching morphogenesis in developing submandibular gland tissue. J Cell Sci.

[42] Fata JE, Mori H, Ewald AJ (2007). The MAPK(ERK-1,2) pathway integrates distinct and antagonistic signals from TGFalpha and FGF7 in morphogenesis of mouse mammary epithelium. Dev Biol.

[43] Yamada A, Futagi M, Fukumoto E (2016). Connexin 43 Is Necessary for Salivary Gland Branching Morphogenesis and FGF10-induced ERK1/2 Phosphorylation. J Biol Chem.

[44] Farooq M, Khan AW, Kim MS (2021). The Role of Fibroblast Growth Factor (FGF) Signaling in Tissue Repair and Regeneration. Cells.

[45] Polito MP, Marini G, Palamenghi M (2023). Decoding the Human Epidermal Complexity at Single-Cell Resolution. Int J Mol Sci.

[46] Li W, Yang J, Cai J (2017). Oil Body-Bound Oleosin-rhFGF-10: A Novel Drug Delivery System that Improves Skin Penetration to Accelerate Wound Healing and Hair Growth in Mice. Int J Mol Sci.

[47] Nishishita N, Ijiri H, Takenaka C (2011). The use of leukemia inhibitory factor immobilized on virus-derived polyhedra to support the proliferation of mouse embryonic and induced pluripotent stem cells. Biomaterials.

[48] Nakao Y, Mitsuyasu T, Kawano S (2013). Fibroblast growth factors 7 and 10 are involved in ameloblastoma proliferation via the mitogen-activated protein kinase pathway. Int J Oncol.

[49] Nie X (2005). Apoptosis, proliferation and gene expression patterns in mouse developing tongue. Anat Embryol (Berl).

[50] Wang J, Sun H, Liu Y (2020). The proliferative and anti-apoptosis functions of KGF/KGFR contributes to bronchial epithelial repair in asthma. Pulm Pharmacol Ther.

[51] Cao L, Chen L, Li H (2019). Differential antigen expression between human eccrine sweat glands and hair follicles/pilosebaceous units. J Mol Histol.

[52] Xu Y, Hong Y, Xu M (2016). Role of Keratinocyte Growth Factor in the Differentiation of Sweat Gland-Like Cells From Human Umbilical Cord-Derived Mesenchymal Stem Cells. Stem Cells Transl Med.

[53] Guo L, Degenstein L, Fuchs E (1996). Keratinocyte growth factor is required for hair development but not for wound healing. Genes Dev.

[54] Jang JH (2005). Stimulation of human hair growth by the recombinant human keratinocyte growth factor-2 (KGF-2). Biotechnol Lett.

[55] Suzuki K, Yamanishi K, Mori O (2000). Defective terminal differentiation and hypoplasia of the epidermis in mice lacking the Fgf10 gene. FEBS Lett.

[56] Lin W, Xiang L, Shi H (2015). Fibroblast growth factors stimulate hair growth through β-catenin and Shh expression in C57BL/6 mice. Biomed Res Int.

[57] Zhang H, Nan W, Wang S (2018). Balance between fibroblast growth factor 10 and secreted frizzled-relate protein-1 controls the development of hair follicle by competitively regulating β-catenin signaling. Biomed Pharmacother.

[58] Rezzola S, Ronca R, Loda A (2019). The Autocrine FGF/FGFR System in both Skin and Uveal Melanoma: FGF Trapping as a Possible Therapeutic Approach. Cancers (Basel).

[59] Thotakura S, Basova L, Makarenkova HP (2019). FGF Gradient Controls Boundary Position Between Proliferating and Differentiating Cells and Regulates Lacrimal Gland Growth Dynamics. Front Genet.

[60] Steinberg Z, Myers C, Heim VM (2005). FGFR2b signaling regulates ex vivo submandibular gland epithelial cell proliferation and branching morphogenesis. Development.

