# Supplementary material for: FGF7 and FGF10 Promote Fate Transition of Human Epidermal Cell-derived Organoids to an Eccrine Gland Phenotype
Source: Int J Biol Sci. 2024 Aug 1;20(11):4162–77. doi: 10.7150/ijbs.97422 (PMC11379064; doi:10.7150/ijbs.97422)
Supplement: Supplementary file 1 — Supplementary figures and tables. [file ijbsv20p4162s1.pdf]

Figure S1. H&E staining of foreskin.

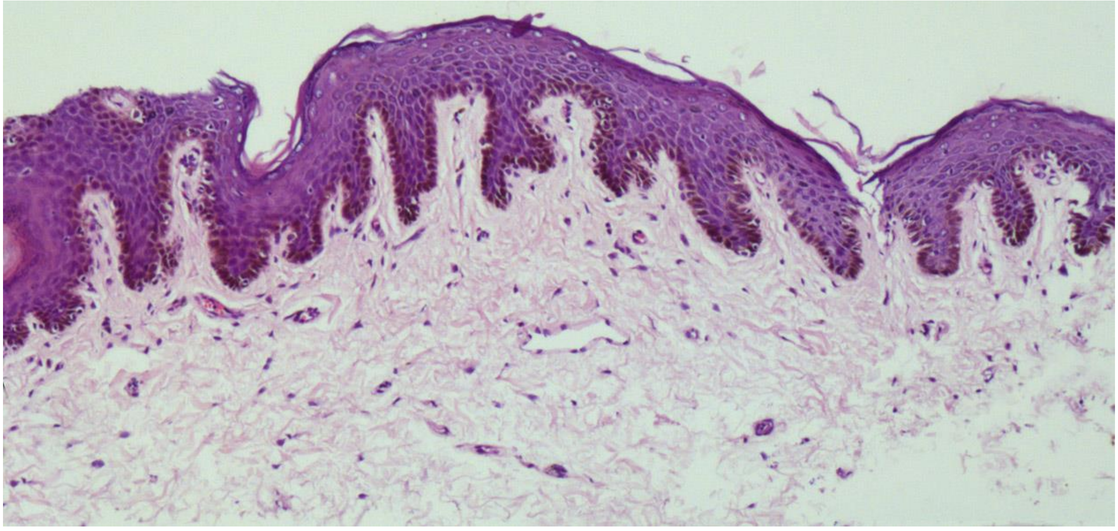

The harvested foreskin was completely devoid of hair follicles, sweat glands, and sebaceous glands.

Figure S2. Enriched signaling pathways of differentially expressed genes.

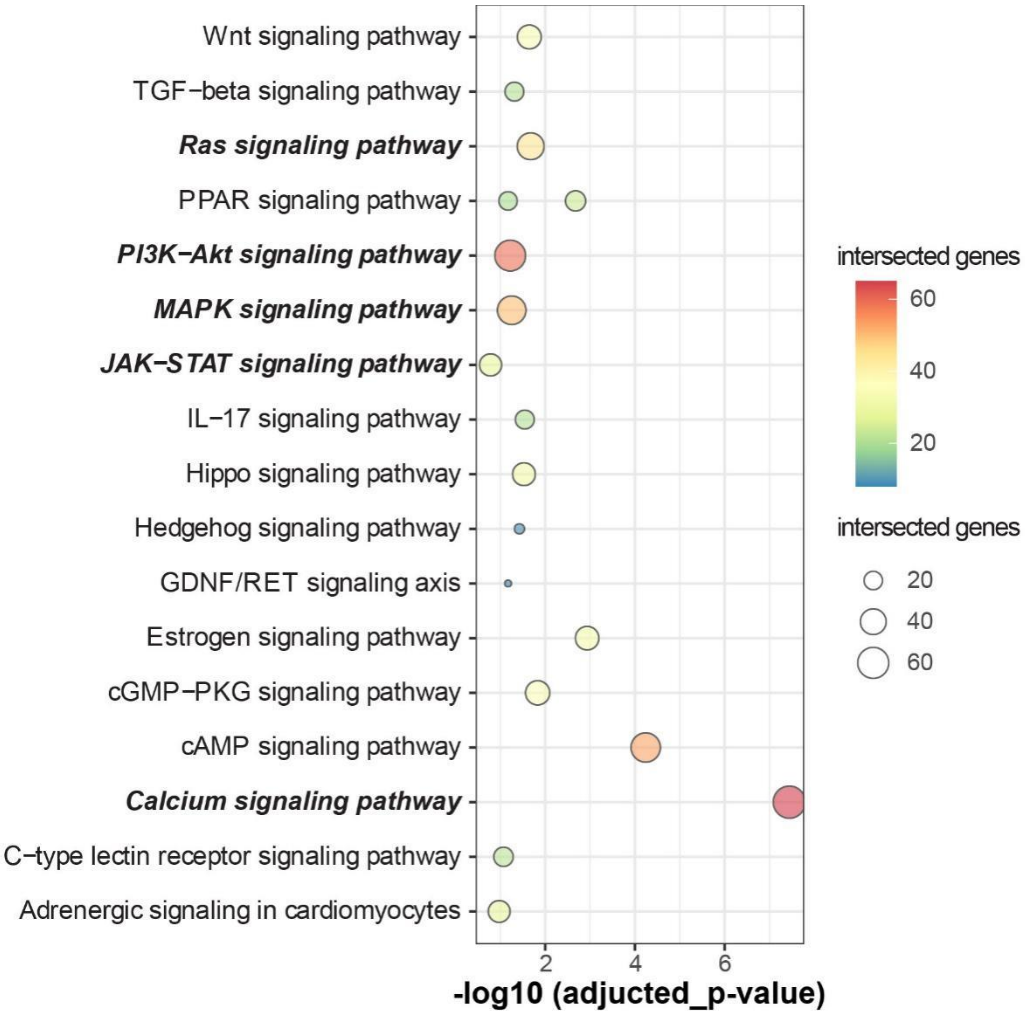

KEGG pathway enrichment analysis showed that up-regulated DEGs were mainly involved in the signaling pathways such as Wnt, TGF-β, RAS, PPAR, PI3K-AKT, MAPK, and JAK-STAT. Among them, RAS, PI3K-AKT, MAPK and JAK-STAT are the downstream signaling pathways regulated by FGF-FGFR.

KEGG, Kyoto encyclopedia of genes and genomes; DEGs, differentially expressed genes; TGF-β, Transforming Growth Factor-β; PPAR, peroxisome proliferator-activated receptor; MAPK, mitogen-activated protein kinas; PI3K, phosphatidylinositol-4,5-bisphosphate 3-kinase; AKT, protein kinase B; JAK, Janus kinase; STAT, signal transducer and activator of transcription;

Figure S3. Morphology of organoids reconstituted by "FDKCs + Matrigel" in an *in vitro* Matrigel plug model.

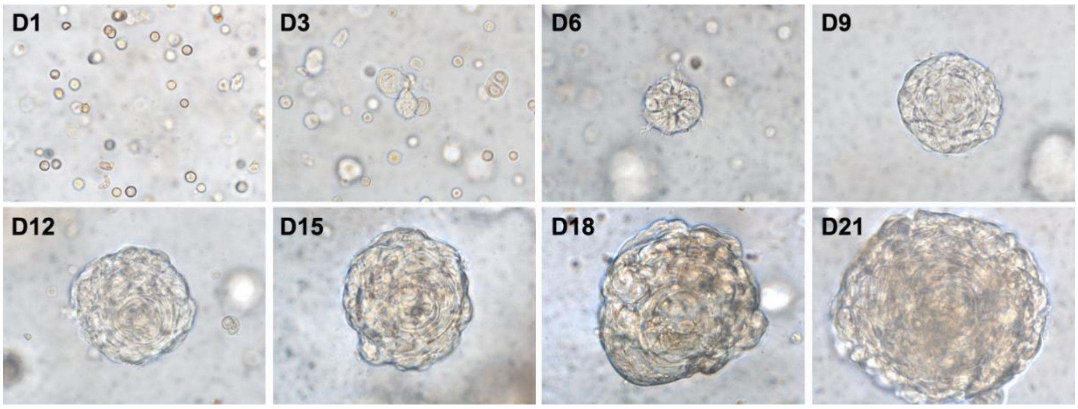

The FDKCs formed small cell clusters 2-3 days post-seeding. The size of the cell clusters increased over time.

Figure S4. Expression of AKT proteins detected by WB.

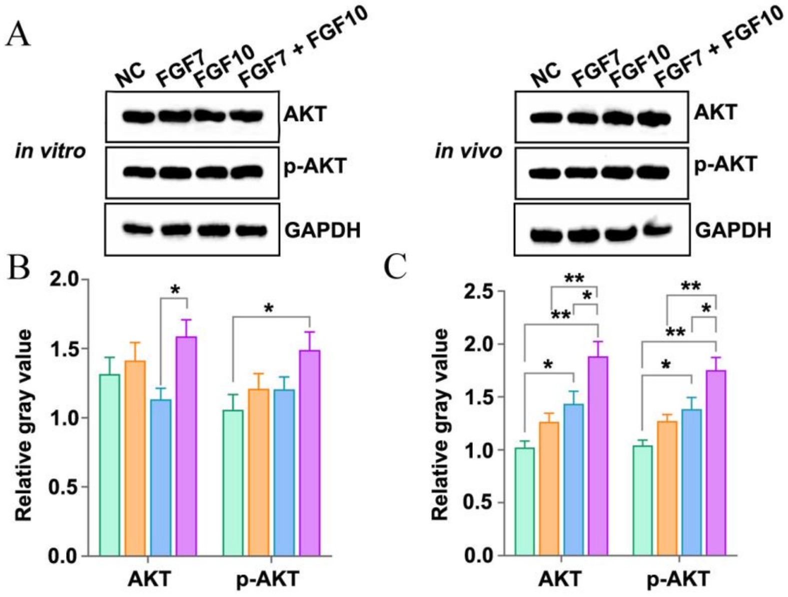

(a, c) Protein expression of AKT and p-AKT in organoids constructed by "FDKCs + Matrigel" in the presence or absence of FGF7 and FGF10.

(b, d) The relative integrated density of AKT and p-AKT in accordance with (a, c), respectively.

Table S1. RT-qPCR Primers.

Prime name	Prime Sequence (5'-3')
Rat-Gapdh-F	CAGTGCCAGCCTCGTCTCAT
Rat-Gapdh-R	AGGGGCCATCCACAGTCTTC
Rat-Hgf-F	GGATTCTTTCAGCCCGGCATCTC
Rat-Hgf-R	ATGGTGACAGGAAGCAGGAGGAG
Rat-Bmp2-F	CGACAGAGTGAAGGCAGATAAGGC
Rat-Bmp2-R	AGGAACGAGCACTGTTAGCGATTC
Rat-Epha2 -F	GGACATCGTCTACAGCGTCACTTG
Rat-Epha2-R	GCCTGAGGTGGTTCTGAGTATTGC
Rat-Wnt5a-F	CAACTGGCGGGACTTTCTCAAGG
Rat-Wnt5a-R	CGGAACTGGTACTGGCACTCTTTG
Rat-Wnt5b-F	CCCTGACTACTGCCTGCGAAATG
Rat-Wnt5b-R	AGCCATCCGTGCCCTCTGAG
Rat-Vwf-F	CTGGTGGAGCCTCTGGTGGTAG
Rat-Vwf-R	CACAAGCCTCCTCCGCAAACC
Rat-Vegfα-F	GTGACAAGCCAAGGCGGTGAG
Rat-Vegfα-R	GATGGTGGTGTGGTGGTGACATG
Rat-Flt1-F	GAGCATCTATCAGGCAGCGGATT
Rat-Flt1-R	GAGCATCTATCAGGCAGCGGATTG
Rat-Fgf3-F	CTTCAAGACCCGCCGCACAC
Rat-Fgf3-R	GCAGCAGCCGTACCATCTCATG
Rat-Fgf7-F	CCGAGCGACACACGAGAAGTTATG
Rat-Fgf7-R	TTCCTCATCTCCTGGGTCCCTTTC
Rat-Fgf10-F	GTCTTCCGTCCCTGTCACCT
Rat-Fgf10-R	TTGTAGCTTCGCACATGCCT
Rat-Fgf22-F	CATCGAGGAGAACGGCTACAACAC
Rat-Fgf22-R	GTCTGCCTTGCCTGGGAATGC
Rat-Shh-F	GAGTGACTGAGGGCTGGGAT
Rat-Shh-R	GTGGTGATGTCCACTGCTCG
Rat-Dkk1-F	AGGTTCCGCAGCCCAGACTC
Rat-Dkk1-R	CGCACAACCGTCATCTCAGAAGG
Human-Gapdh-F	GAGTCAACGGATTTGGTCGT
Human-Gapdh-R	TTGATTTTGGAGGGATCTCG
Human-Erk-F	ATGGTGTGCTCTGCTTATGATA
Human-Erk-R	TCTTTCATTTGCTCGATGGTTG
Human- Jnk-F	ACACCACAGAAATCCCTAGAAG
Human-Jnk-R	CACAGCATCTGATAGAGAAGGT
Human-p38-F	ATTTCAGTCCATCATTCATGCG
Human- p38-R	GTAAAAACGTCCAACAGACCAA
Human-Fgfr1-F	TGGAGTTAATACCACCGACAAA
Human-Fgfr1-R	GATGATGATCTCCAGGTACAGG
Human-Fgfr2-F	CTAAAGGCAACCTCCGAGAATA
Human-Fgfr2-R	ACATTTTTGGGAAGCCAAGTAC
Human-Krt7-F	AGTATGAGGAGATGGCCAAATG
Human-Krt7-R	CTGGTTCTTGATGTTGTCGATC
Human-Krt73-F	AGCTGACCCGTCTCATCCAA
Human-Krt73-R	GCTTCACGCTCAAAAGCTCTTG

Mouse-beta-Actin-F	CCTCACTGTCCACCTTCCA
Mouse-beta-Actin-R	GGGTGTAAAACGCAGCTCA
Mouse-Dkk1-F	GGTTCTTGGCCGTGTTT
Mouse-Dkk1-R	AGGTTCTTGATCGCGTTG
Mouse-Vegfα-F	CCTTCAGCTCGCTCCTC
Mouse-Vegfα-R	CCTTCAGCTCGCTCCTC
Mouse-Hgf-F	GACCCTGACACCCCTTG
Mouse-Hgf-R	GTATTGCTGGTTCCCCTGT
Mouse-Flt1-F	AGATGTGCCGAATGGCT
Mouse-Flt1-R	TGTCCGTAGCAGAATCCAG
Mouse-Vwf-F	AGGCCAATCGCACTTCA
Mouse-Vwf-R	ACAGCATCAGGGTCATCG
Mouse-Epha2-F	GCCAGTTTAGCCACCACAA
Mouse-Epha2-R	TGCGATACCCCTCAGCA
Mouse-Wnt5a-F	GATGCTGGCCCTCTGTG
Mouse-Wnt5a-R	ATCCCCACCTGTCTCCTG
Mouse-Wnt5b-F	CAAGAGCGTTCAGGTGGA
Mouse-Wnt5b-R	TTGTGAGGCGGAGAGGA
Mouse-Fgf3-F	CAGAGTAGCCAGCCACGA
Mouse-Fgf3-R	AGCCAGTCCACCTGTATGC
Mouse-Fgf7-F	CTAGCTTGCAATGACATGAGTC
Mouse-Fgf7-R	TCCATGATGTTGTAGCTGTTCT
Mouse-Fgf10-F	ATGTCCGCTGGAGAAGG
Mouse-Fgf10-R	ACGGCAACAACTCCGAT
Mouse-Fgf22-F	CCGTTCTGTCCGTGTGG
Mouse-Fgf22-R	GTAGACCCGCGACCCAT
Mouse-Shh-F	CTTGCTTCCTCGCTGCTGGTG
Mouse-Shh-R	AGGGTCTTCTCGGCTACGTTGG
Mouse-Bmp2-F	CCACCCCAAGACACAGTT
Mouse-Bmp2-R	GCACGTCCATTGAGAGAGT

Gadph, glyceraldehyde-3-phosphate dehydrogenase; Erk1/2, extracellular regulated protein kinases1/2; Jnk, c-Jun N-terminal kinase; Fgf, fibroblast growth factor; Fgfr, fibroblast growth factor receptor; Bmp2, bone morphogenetic protein 2; Vegfα, vascular endothelial growth factor α; Epha2, erythropoietin-producing hepatocellular receptor A2; Vwf, von Willebrand factor; Flt1, vascular endothelial growth factor receptor 1; Hgh, hepatocyte growth factor; Dkk1, Dickkopf-1; Shh, sonic hedgehog; Krt, keratin**.**

Table S2. Summary of primary antibodies and their application and dilution.

Primary antibodies	Manufacturer	ICC Dilution	IHC Dilution	WB Dilution
Rabbit polyclonal anti-K7 (ab181598)	Abcam, Cambridge, UK	-	1:1000	1:2000
Rabbit polyclonal anti-K27 (ab220725)	Abcam, Cambridge, UK	-	1:1000	-
Rabbit monoclonal anti-K73 (ab181383)	Abcam, Cambridge, UK	-	1:500	1:2000
Mouse monoclonal anti-NKA (sc-58628)	Santa Cruz, CA, USA	-	1:500	-
Rabbit monoclonal anti-FGFR1 (9740)	Cell signaling, MA, USA	-	-	1:1000
Rabbit monoclonal anti-FGFR2 (23328)	Cell signaling, MA, USA	-	-	1: 1000
Rabbit monoclonal anti-ERK1/2 (4695)	Cell signaling, MA, USA	-	-	1:2000
Rabbit monoclonal anti-p-ERK1/2 (4370)	Cell signaling, MA, USA	-	-	1:2000
Rabbit monoclonal anti-p38 (8690)	Cell signaling, MA, USA	-	-	1:2000
Rabbit monoclonal anti-p-p38 (4511)	Cell signaling, MA, USA	-	-	1:2000
Rabbit polyclonal anti-JNK (9252)	Cell signaling, MA, USA	-	-	1:2000
Mouse monoclonal anti-p-JNK (9255)	Cell signaling, MA, USA	-	-	1:2000
Rabbit anti-GAPDH (ab181602)	Abcam, Cambridge, UK	-	-	1:5000
Rabbit monoclonal anti-AKT (4691)	Cell signaling, MA, USA	-	-	1:2000
Rabbit monoclonal anti-p-AKT (4060)	Cell signaling, MA, USA	-	-	1:2000
Rabbit monoclonal anti-K5 (ab52635)	Abcam, Cambridge, UK	1:100	-	-
Rabbit monoclonal anti-K14 (ab181595)	Abcam, Cambridge, UK	1:1000	-	-
Mouse monoclonal anti-alpha 6-integrin (ab20142)	Abcam, Cambridge, UK	1:50	-	-
Mouse monoclonal anti-beta 1-integrin (ab30394)	Abcam, Cambridge, UK	1:50	-	-

ERK1/2, extracellular regulated protein kinases1/2; p-ERK, phospho-ERK1/2; JNK, c-Jun N-terminal kinase; p-JNK, phospho-JNK; p-P38, phospho-P38; FGF, fibroblast

growth factor; FGFR, fibroblast growth factor receptor; GADPH, glyceraldehyde-3-phosphate dehydrogenase; NKA, Na+-K+-ATPase; ICC, immunocytochemistry; IHC, immunohistochemistry; WB, western blotting; AKT, protein kinase B; p-AKT, phospho-AKT.

Table S3. Summary of secondary antibodies and their application and dilution.

**Secondary antibodies**	**Manufacturer**	**ICC Dilution**	**IHC Dilution**	**WB Dilution**
Alexa Fluor 488-labeled goat anti-Mouse IgG(H+L) (A0428)	Beyotime, Shanghai, China	1:500	1:500	-
Alexa Fluor 488-labeled goat anti-Rabbit IgG(H+L) (A0423)	Beyotime, Shanghai, China	1:500	1:500	-
Cy3-labeled goat anti-Mouse IgG(H+L) (A0521)	Beyotime, Shanghai, China	-	1:500	-
Cy3-labeled goat anti-Rabbit IgG(H+L) (A0516)	Beyotime, Shanghai, China	-	1:500	-
HRP-labeled goat anti-Mouse IgG(H+L) (ab2467)	Abcam, Cambridge, UK	-	-	1:5000
HRP-labeled goat anti-Rabbit IgG(H+L) (ab6789)	Abcam, Cambridge, UK	-	-	1:5000
